# The type IV pilin, PilA, is required for full virulence of *Francisella tularensis *subspecies *tularensis*

**DOI:** 10.1186/1471-2180-10-227

**Published:** 2010-08-26

**Authors:** Anna-Lena Forslund, Emelie Näslund Salomonsson, Igor Golovliov, Kerstin Kuoppa, Stephen Michell, Richard Titball, Petra Oyston, Laila Noppa, Anders Sjöstedt, Åke Forsberg

**Affiliations:** 1 CBRN Defence and Security, FOI Swedish Defence Research Agency, 901 82 Umeå, Sweden; 2 Umeå Centre for Microbial Research (UCMR) and Laboratory for Molecular Infection Medicine (MIMS), Sweden, Department of Molecular Biology, Umeå University, 901 87 Umeå, Sweden; 3 Umeå Centre for Microbial Research (UCMR) and Laboratory for Molecular Infection Medicine (MIMS), Sweden, Department of Clinical Microbiology, Umeå University, 901 85 Umeå, Sweden; 4 School of Biosciences, University of Exeter, Devon, EX4 4QD, UK; 5 Defence Science and Technology Laboratory, Porton Down, Salisbury, Wiltshire SP4 OJQ, UK

## Abstract

**Background:**

All four *Francisella tularensis *subspecies possess gene clusters with potential to express type IV pili (Tfp). These clusters include putative pilin genes, as well as *pilB*, *pilC *and *pilQ*, required for secretion and assembly of Tfp. A hallmark of Tfp is the ability to retract the pilus upon surface contact, a property mediated by the ATPase PilT. Interestingly, out of the two major human pathogenic subspecies only the highly virulent type A strains have a functional *pilT *gene.

**Results:**

In a previous study, we were able to show that one pilin gene, *pilA*, was essential for virulence of a type B strain in a mouse infection model. In this work we have examined the role of several Tfp genes in the virulence of the pathogenic type A strain SCHU S4. *pilA*, *pilC*, *pilQ*, and *pilT *were mutated by in-frame deletion mutagenesis. Interestingly, when mice were infected with a mixture of each mutant strain and the wild-type strain, the *pilA, pilC *and *pilQ *mutants were out-competed, while the *pilT *mutant was equally competitive as the wild-type.

**Conclusions:**

This suggests that expression and surface localisation of PilA contribute to virulence in the highly virulent type A strain, while PilT was dispensable for virulence in the mouse infection model.

## Background

*Francisella tularensis *is a Gram-negative facultative intracellular bacterial pathogen and the causative agent of tularemia. Infections have been reported in a range of vertebrates as well as invertebrates [1]. Natural infections can occur via ingestion of contaminated food or water, handling of infected animals, bites by infected arthropods, including mosquitoes and ticks, or via inhalation [2]. *F. tularensis *is divided into four subspecies, where ssp. *holarctica *(type B) is most widely spread and found in the major part of Europe, Asia, and North America. *F. tularensis *ssp. *tularensis *(type A) is found exclusively in North America and ssp. *mediasiatica *in Central Asia. Finally, ssp. *novicida *has been isolated in several locations in North America, as well as in Australia [3,4]. Human infections are mainly caused by type A or type B strains, where type A strains are significantly more virulent than type B strains. Our knowledge regarding virulence determinants in *F. tularensis *is rather limited. However, available genome information [5,6] together with development of genetic tools [7], has resulted in increased understanding of the molecular mechanisms of *F. tularensis *infections.

The genome of *F. tularensis *encodes gene clusters involved in secretion and assembly of type IV pili (Tfp) [5]. Tfp are complex adhesins involved in important host cell interactions for human pathogens like *Neisseria *spp., *Pseudomonas aeruginosa *and *Vibrio cholerae *[8-11]. The pilus fiber is composed of one major pilin subunit and several additional minor pilins required for function and/or assembly of the pilus [12,13]. However, the exact roles of the minor pilins are still not completely understood. The pilus is translocated to the cell surface via the secretin, PilQ, which forms a pore in the outer membrane through which the pilus is transported and extended [14]. PilD is a peptidase cleaving the prepilin subunits [11] and PilC is a transmembrane protein spanning across the plasma membrane [15]. Furthermore, two ATPases, PilB and PilT, are involved in extension and retraction, of the pilus [16,17]. In some bacteria Tfp can mediate twitching motility, an activity that is PilT dependent [18].

There is evidence that *F. tularensis *expresses Tfp-like surface structures on the bacterial surface [19-21], and the putative pilin, PilA, has been shown to be required for virulence of type B strains in a mouse infection model [22]. Interestingly, due to direct repeat mediated deletion, the *pilA *gene has been lost in the attenuated live vaccine strain LVS [22,23], supporting the significance of PilA for virulence [24]. There are also other potentially significant differences between different *F. tularensis *subspecies. In ssp. *novicida *that is non-pathogenic for humans, PilA differs in the amino acid sequence compared to the virulent type A strain SCHU S4 [25]. On the contrary, *pilA *of virulent type B strains is essentially identical to the corresponding gene in type A strains, however, several other differences are apparent between the two subspecies. Two predicted pilin genes, *pilE *and *pilV*, and the ATPase encoding gene, *pilT*, are pseudogenes in type B strains [19,21,22,26]. These differences are likely to be significant since *pilA *has been linked to virulence of type B strains and is missing in the attenuated vaccine strain LVS. Furthermore, PilA of ssp. *novicida *was recently shown to be involved in protein secretion that was coupled to Tfp [20,25]. Interestingly, mutation of *pilA *and loss of protein secretion resulted in increased virulence in a mouse infection model [25]. As the human pathogenic type A and type B strains do not secrete detectable levels of proteins *in vitro*, it is possible that one step in the evolution of human pathogenic variants of *F. tularensis *from ssp. *novicida *has involved loss of protein secretion as a consequence of changes in PilA structure and function.

In this work we wanted to address the question if PilA is involved in virulence of the highly pathogenic type A strain SCHU S4, similarly to what we have previously shown for type B strains, and if Tfp secretion and assembly genes are required for virulence.

## Results

### Construction of non-polar pilin gene mutants

In a recent study, we were able to demonstrate that the *pilA *gene can be lost by a deletion event mediated by direct repeats flanking the gene [22]. Type B strains lacking *pilA *were found to be attenuated for virulence in a mouse infection model. In this study we wanted to extend this work to the highly pathogenic type A strain SCHU S4, and therefore we constructed a specific *pilA *deletion mutant using our previously described allelic exchange technique [7]. In addition, to address the significance of secretion and assembly of PilA, we also engineered in-frame deletions in *pilC *and *pilQ*, encoding a transmembrane protein and a secretin, respectively. For some pathogens, Tfp expression is associated with a unique ability to retract the pili, a phenotype depending on the ATPase PilT. Interestingly, *pilT *appears to be functional in type A strains, while it is a pseudogene in the less pathogenic type B strains. In order to elucidate if the expression of PilT could be correlated to the higher virulence of type A strains, we also constructed an in-frame deletion in the *pilT *gene.

**Figure 1:**
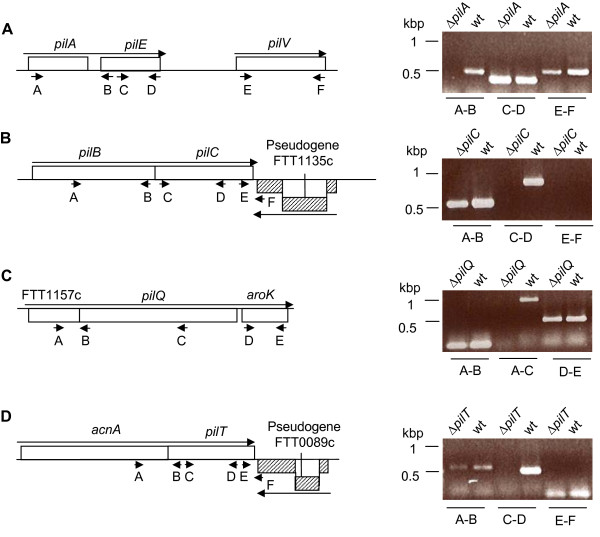
**A-D. Analysis of gene transcription in wild-type and mutant strains of *F. tularensis *using RT-PCR of mRNA**. The location of the different primers used is indicated by small arrows. Large arrows illustrate direction of transcription. Control reactions where reverse transcriptase was omitted were all negative (data not shown).

In order to verify that the mutations did not have a major impact on neighboring gene transcription, each region was analysed by RT-PCR on mRNA extracted from the mutant strains and compared to the isogenic wild-type strain (Fig. 1). Thereby we could confirm that none of the deletion events caused any polar effects on transcription. Both *pilC *and *pilT *are flanked by pseudogenes situated directly downstream of each gene that were found not to be transcribed neither in the wild-type nor in the *pilC *or *pilT *mutant strains. The upstream genes of *pilC *and *pilT *were readily transcribed at similar levels in the wild-type and mutant strains. In the case of the *pilQ *mutant, we could verify non-polarity on the downstream *aroK *gene.

We also attempted to make an in-frame deletion of the *pilA *gene, but in spite of several attempts we were unable to generate an unmarked deletion. It is possible that this is linked to the fact that there are two direct repeats flanking *pilA *and that this somehow affects the recombination in this region [22]. We therefore chose a different strategy where we introduced a chloramphenicol resistance gene to allow for direct selection of the mutational event. In order to lower the risk of polar effects on the downstream *pilE *gene the resistance gene was inserted in the same orientation as the *pilAE *genes. We could also verify that the levels of *pilE *transcription were similar in the *pilA *mutant and wild-type strain, suggesting that there were no major polar effects on downstream genes. We have previously shown that *pilV *is transcribed from a promoter downstream of *pilE *gene in type strains [22] and also in this case *pilV *transcription levels were similar in the *pilA *and the wild-type strain. From this we conclude that none of the mutations generated any major polar effect on transcription of neighboring genes.

### PilA expression in Tfp mutant strains

**Figure 2:**
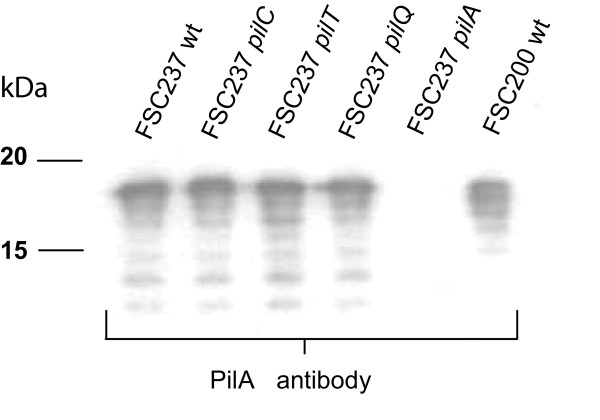
**PilA is expressed at wild-type levels in all strains, except for the *pilA *deletion mutant**. Different pili mutants in the *F. tularensis *strain SCHU S4, analysed by Western blot using an anti-PilA antiserum. Lane; 1, FSC237 (SCHU S4, Type A); 2, FSC237 *pilC *deletion mutant; 3, FSC237 *pilT *deletion mutant; 4, FSC237 *pilQ *deletion mutant; 5, FSC237 *pilA *deletion mutant; 6, FSC200 (Type B).

Next we wanted to address if any of the mutations influenced PilA expression. Therefore the expression of PilA in the different mutant strains was analysed by Western blot analysis. All mutants, except for *pilA*, expressed PilA at levels similar to the isogenic wild-type strain SCHU S4 (Fig. 2). The apparent molecular mass of PilA was similar to what has previously been shown for type B strains, 4-5 kDa larger than expected from their calculated molecular masses, indicating PilA to be post-transcriptionally modified, presumably by glycosylation [22]. Thus, with the exception of the *pilA *mutant, all the mutants expressed PilA at similar levels as the wild-type strain SCHU S4.

### *pilA*, *pilC *and *pilQ *contribute to virulence of SCHU S4

**Figure 3:**
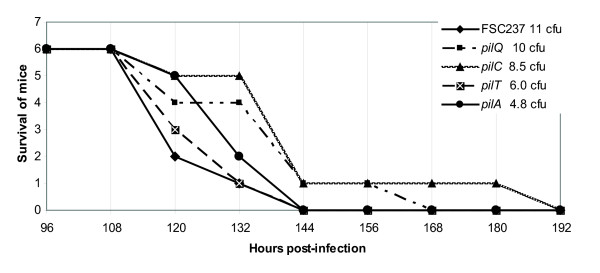
**Infection kinetics are slightly delayed for mice infected with the *pilA, pilC*, and *pilQ *deletion strains**. Groups of six mice were infected via the subcutaneous route and the survival followed over time. The exact dose for each strain was determined by retrospective viable count.

When we studied the role of *pilA *in LVS we could establish that the pilin had a major impact on virulence [24]. More recently, we have also made a specific *pilA *mutant in a recent clinical type B isolate. In this highly virulent type B strain, the attenuation seen for the *pilA *mutant was less marked, but still the lethal infection dose for the mutant was about 40-fold higher compared to the isogenic wild-type strain (unpublished data). Here, we wanted to address the role of *pilA*, the assembly/secretion genes, *pilC *and *pilQ*, and the ATPase encoding gene *pilT*, in the virulence of the highly pathogenic type A strain SCHU S4. First we verified that the growth kinetics *in vitro *was not affected in the mutant strains by measuring growth in liquid medium (data not shown). In order to evaluate the importance of the pili genes for *in vivo *virulence, mice were infected via the subcutaneous (s.c.) route with the mutant strains, as well as the isogenic wild-type strain SCHU S4. We used the s.c. route of infection as it can be more discriminative than the intraperitoneal (i.p.) route of infection. For instance, the attenuated vaccine strain LVS is still virulent by the i.p. route but highly attenuated by the s.c. route of infection in mice. Two different infection doses were used; approximately 10 and 100 bacteria respectively. Groups of six mice were infected with each dose and the progress of the infection was monitored over time. Small differences in infection kinetics were observed for the *pilA, pilC *and *pilQ *mutants, and mice infected with these strains showed a slightly delayed time to death compared to mice infected with the wild-type strain. Still, as SCHU S4 is very virulent in mice, even at the lowest doses (5 - 10 bacteria), all animals had succumbed to the infection after six to eight days post infection, making it difficult to establish the significance of the result (Fig. 3, Table 1). Therefore, we decided to perform competitive infections between the wild-type strain and the different isogenic mutants. In this case all mutants, except *pilT*, were out-competed by the wild-type strain SCHU S4. For the *pilA, pilC *and *pilQ *mutant strains, the ratios were 0.14, 0.34 and 0.16 (Table 1), respectively, suggesting PilA to be a virulence determinant also in the type A strain SCHU S4. The fact that the ratio was similar for the *pilC *and *pilQ *mutants indicate that assembly/surface localisation of the pilin PilA is required for full virulence in the mouse infection model. Statistical analysis verified that the differences in ratios for these three mutants were significant at *P *< 0.05 (data not shown). Somewhat surprisingly, the *pilT *mutant was not out-competed by the wild-type strain in the mixed infection experiment. The ratio (1.98) suggests that PilT is dispensable for virulence in the subcutaneous mouse infection model (Table 1). In this case the higher ratio for the pilT mutant was not statistically significant (data not shown).

**Table 1:** Mice infection data.

SCHU S4	Infection dose (cfu)	CI value
wt	11	
*pilA*	4.8	0.14
*pilC*	8.5	0.34
*pilT*	6.0	1.98
*pilQ*	10	0.16

Infection dose (cfu) in a standard infection study, and CI value in a competitive index assay.

## Discussion

Recently, we have provided evidence that PilA, one of the type IV pilins encoded by the human pathogenic subspecies of *F. tularensis*, is an important virulence determinant for type B strains [22]. In addition, we have established that loss of the *pilA *gene is one of two major genetic events, responsible for the attenuation of the live vaccine strain, LVS [6,24]. Even though we have been able to demonstrate PilA to be both surface located in *F. tularensis *[22] and able to form functional Tfp in the heterologous system in *Neisseria gonorrhoeae *[27], we have still not managed to verify PilA to be an actual structural component of Tfp expressed by *F. tularensis*.

In this study, we present evidence that PilA and the Tfp assembly/secretion proteins, PilC and PilQ, are required for full virulence of the type A strain, SCHU S4, the most virulent subspecies of *F. tularensis*. In infections with individual mutants, we were unable to show that mutations of the putative Tfp genes resulted in a significant attenuation. However, when we conducted mixed infections, where the ability of the mutants to compete with the wild-type strain was assessed, it became more obvious that Tfp encoding genes may play a role in the virulence of SCHU S4. This is in line with our observation that *pilA *mutants in highly virulent clinical isolates of type B strains are less attenuated compared to type B strains with weaker virulence, like LVS [22,24]. A general problem with the mouse infection model is that mice are highly susceptible to *Francisella *and do not discriminate between the virulence properties of different *F. tularensis *subspecies in the same way as the human infection. The emerging picture is that *pilA *mutants show less attenuation in the most pathogenic subspecies. Still, we believe that PilA, and potentially also Tfp, may play an important role in virulence. This theory is supported by the fact that LVS has lost *pilA*, and that this is one of the causes of its attenuation [24]. When genomes of different subspecies are compared, one striking difference is that the *pilT *gene is a pseudogene in type B strains, due to a point mutation introducing a stop codon in the middle of the gene [26]. Interestingly, in a study involving the attenuated type B strain LVS the *pilT *gene was demonstrated to be involved in pili assembly, adherence and virulence [19]. Chakraborty with colleagues have suggested the possibility that the truncated PilT protein somehow has retained function in LVS [19]. Their findings are somewhat surprising since in other Tfp expressing pathogens the PilT protein is only involved in pilus retraction and not in pilus assembly. The *pilT *mutant in SCHU S4 did not have any impact on the virulence in the subcutaneous mouse infection model. However, the fact that *pilT *is intact in most pathogenic type A strains suggests that PilT might, at least partly, contribute to the higher virulence of type A strains. As mentioned above, this infection model has limitations and may not reflect virulence in humans. Another possibility is that PilT may rather play a role in the environment and/or in transmission of tularemia than in the animal/human infection. With the genetic tools and the availability of specific mutants in the Tfp encoding gene clusters of SCHU S4, it will now be possible to address the role of the Tfp system in other infection models, for survival in the environment, and perchance for vector-borne transmission.

## Conclusions

We have shown that *pilA *is required for full virulence of SCHU S4 in mice - a result in line with our earlier findings in type B strains. In addition, we have also demonstrated that the pilin assembly genes, *pilC *and *pilQ*, are needed for full virulence of SCHU S4. An unexpectedly finding is that PilT, even though it is functional only in type A strains, did not contribute to virulence in the mouse subcutaneous infection model.

## Methods

### Bacterial strains, plasmids, growth conditions, and DNA methods

The bacterial strains and plasmids used in this study are listed in Table 2. *F. tularensis *strains were grown on modified Thayer-Martin agar or Blood Cystine Glucose agar (BCGA) at 37ºC in 5% CO_2_. *Escherichia coli *strains were grown on blood agar base (BAB; Merck) plates or in Luria Bertani broth (LB). Antibiotics were used at the following concentrations: kanamycin 50 μg/ml and chloramphenicol 2.5 μg/ml (*F. tularensis*), or 25 μg/ml (*E. coli*). Preparation of plasmid DNA, restriction enzyme digests, ligations and transformations into *E. coli *were performed essentially as described [28]. Generally, the primers (Table 3) were constructed based on the genomic information from the FSC237 (SCHU S4) and FSC155 (LVS) genomes. The amplified PCR fragments were first cloned into the pCR^®^4.0-TOPO cloning vector (Invitrogen AB, Stockholm, Sweden), sequenced by Eurofins MWG Operon, and subsequently cloned into the suicide vectors pSMP22 [29] or pDM4 [30].

**Table 2:** Strains and plasmids used in this study

Strains	Genotype/phenotype	Source
*F. tularensis*		
FSC237	*tularensis; *SCHU S4	Human ulcer 1941, Ohio
	FSC237*; *Δ*pilA; *deletion of codons 1-135	This study
	FSC237*; *Δ*pilC *(FTT1134); in frame deletion of codons 5-405	This study
	FSC237*; *Δ*pilQ *(FTT1156); in frame deletion of codons 13-593	This study
	FSC237*; *Δ*pilT *(FTT0088); in frame deletion of codons 7-336	This study
*E. coli*		
Top10	F^- ^*mcrA *Δ(*mrr-hsdRMS-mcrBC*), Φ80*lacZ*ΔM15 Δ*lacX74 recA1 deoR araD139 *(Δ*ara-leu*)*7697 galU galK rpsL *(Sm^r^) *endA1 nupG*	Invitrogen
S17-1Λpir	*recA, thi, pro, hsdR*^-^*M*+,<RP4:2-Tc:Mu:Km:Tn*7*> Tp^R^, Sm^R^	[32]

**Plasmids**		

pCR^®^4.0	TOPO-cloning vector. Amp^R^, Km^R^	Invitrogen
pDM4	Suicide plasmid. sacB; mobRP4; oriR6K; Cm^R^	[30]
pSMP22	Suicide plasmid. *groESL *promoter, ori T, bla, sacB	[29]
pSMP50CAM	432 bp fragment of *pilA *including a chlorampenicol resistance cassette cloned into pSMP22. Cm^R^	This study
pAL12	2072 bp fragment of approximately 1 kb upstream and 1 kb downstream of *pilC *cloned in XbaI and SalI site of pDM4. Cm^R^	This study
pAL16	2122 bp fragment of approximately 1 kb upstream and 1 kb downstream of *pilQ *cloned in XbaI and SalI site of pDM4. Cm^R^	This study
pAL18	2133 bp fragment of approximately 1 kb upstream and 1 kb downstream of *pilT *cloned in XbaI and SalI site of pDM4. Cm^R^	This study

**Table 3:** Primers used in this study

Primer	Primer sequence 5'-3'	RE site
pilA LFF	GAGCTCACGCGT-CTTACTTGCCGGATCATTACCAAC	SphI
pilA LFR	CTGCAG-CCTTCTTTATAGTTTAGTTTAC	PstI
pilA RFF	CTGCAGGTAGATAAACTAAGCCACTTTCATGTG	PstI
pilA RFR	GGATCCGCATGCTCAAGGCTTCTGTCAATCTTGTTC	MluI
CAM PstIF	GCCTGCAGGTAAGAGGTTCCAACTTTCAC	PstI
CAM PstIR	TGATCTGCAGTTACGCCCCGCCCTGCCACTCATC	PstI
PilC-A	GCATGTCCTAGGGTCAAGCTTAGATATTGCTGAA	AvrII
PilC-B	TATATCGCATCGCCAAATAGCATATTTTTTATTCC	
PilC-C	GCTATTTGGCGATGCGATATAATATACTTTTAAAAA	
PilC-D	GCATGTGTCGACGTCCTGAGAAAATATCTAGACA	SalI
PilT-A	CATTATGTCGACTATGCAACAGTTCTTGATGGT	SalI
PilT-B	TACTACAATGTATAGTAATTTTCTTATCATATCAAG	
PilT-C	AGAAAATTACTATACATTGTAGTAAGGTAATCA	
PilT-D	CATTATTCTAGACAGGATTAACGGCAGCTAAAA	XbaI
PilQ-A3	GCATGTCCTAGG TCAGTCAATGGAAGCACAGAT	AvrII
PilQ-B3	TATCTGCTATCATGTTAGAACAACTAATAACTTCTT	
PilQ-C3	TTGT TCTAACATGATAGCAGATAATAGTTGCAAA	
PilQ-D3	GCATGTGTCGACAGAAAGTAATGTTGTTGGTATTT	SalI
**RT-PCR** **primers**		
PilA_A	GATCCCGATGTACTCTAACTA	
PilA_B	CCATTAGCTCAACTAGTGAGAA	
PilA_C	ATCTTAGCAGCTGTAGCAATA	
PilA_D	GGGGTAGTACTTTAAATCCT	
PilA_E	CTTACTGAGTTACTTGTTGTTAT	
PilA_F	GTCTTTCTGATCTATATGCTTC	
PilC_A	GTCAAGCTTAGATATTGCTGAA	
PilC_B	GTCTCTGGAGCACTGTTTGTAT	
PilC_C	AAGGTAGTATTGATGCTGACAC	
PilC_D	CCGTTGCTAAAGACACCATA	
PilC_E	GATGCGATATAATATACTTTTAAAAA	
PilC_F	CGAATTGGTATTGGCCAGAT	
PilQ_A	TATGGTCAGGTAGAAGATGTAA	
PilQ_B	CATCAATTTACCTTACTAATGTAT	
PilQ_C	GCCTGAGCAGTAGTATAGTTT	
PilQ_D	AGTTGGTGCTGGAAAATCTAC	
PilQ_E	CAGGATAGTTTCTTCTACTAAA	
PilT_A	CTATTAGGCGTGAAAGCAGTT	
PilT_B	TAGTAATTTTCTTATCATATCAAG	
PilT_C	ATGATGCGAGATTTAGGGTA	
PilT_D	CAGCAGGTGGAAATACAGAT	
PilT_E	TACATTGTAGTAAGGTAATCA	
PilT_F	GGTAGAGTTGAATCAGCGTTTA	

### Construction of deletion mutants of *pilA, pilC*, *pilQ*, and *pilT *in FSC237

Left and right flanking regions of *pilA *(FTT0890c, SCHU S4 nomenclature) were PCR amplified using the primer pairs pilA_LFF/pilA_LFR and pilA_RFF/pilA_RFR, and cloned into pGEMT-easy (Promega). The left flank was excised with EcoRI and PstI and the right flank was excised with BamHI and PstI. The fragments were ligated into an EcoRI/BamHI digested pBluescript KS+ vector (Stratagene), giving rise to pSMP47. A chloramphenicol resistance gene was PCR amplified from pDM4 with the primer pair CAM_PstIF/CAM_PstIR, digested with PstI, and cloned into pSMP47, generating pSMP48 containing the left and right flanks of *pilA *disrupted by a chloramphenicol cassette. The mutated allele of *pilA *was excised from pSMP48 with SphI and MluI, cloned into pSMP22, and the resulting plasmid pSMP50CAM (Table 2) was introduced into strain FSC237 by conjugal mating as previously described [7].

The deletion constructs for *pilC, pilQ *and *pilT *were obtained by overlapping PCR using the following primer pairs PilX_A, PilX_B, PilX_C and PilX_D (Table 3). For the deletion constructs of *pilC *and *pilQ *strain FSC237 was used as template and for the *pilT *deletion the strain FSC155 was used as a template. The sequence for the *pilT *construct is almost identical between FSC155 and FSC237 except for three substitutions upstream of the deletion in non-coding sequences, and eight substitutions in a downstream pseudogene. The PCR fragments were cloned into the suicide vector pDM4 and the resulting plasmids pAL12 (*pilC*), pAL16 (*pilQ*), and pAL18 (*pilT*) (Table 2) were introduced into strain FSC237 by conjugal mating as previously described [7]. The *in vitro *growth rate of the different mutant strains were compared with the wild type strain by measuring OD at different time points, 0 h, 6 h and ON after dilution in Chamberlain medium.

### RNA isolation and RT-PCR

Bacteria were grown for 18 h on plates, harvested and suspended in TRIzol reagent (Life Technologies). Total RNA was extracted and treated with RNase-free DNase I (Roche), phenol extracted, and precipitated by ethanol. An aliquot of the RNA (3 μg) was used to synthesize cDNA using random hexamers (final concentration 25 ng/μl) and Superscript III reverse transcriptase as described by the manufacturer (Life Technologies). In control experiments samples processed without addition of RT enzyme were used.

### Animal infections

*F. tularensis *strains were grown for 16 h on BCGA before the bacteria were suspended in phosphate buffered saline (PBS) pH 7.4 to an OD_540 _= 1, which normally corresponds to approximately 2 × 10^9 ^bacteria/ml. The bacterial suspension was then diluted in PBS into two doses used for challenge, around 10 and 100 bacteria in a total volume of 100 μl. All bacterial infections were initiated by subcutaneous injections of 6-8 week old C57Black/6 female mice. The study was approved by the Local Ethical Committee on Laboratory Animals in Umeå, Sweden. For competitive index (CI) infections, the mice were infected with a 50:50 mixture of mutant and wild-type strains with around 50 bacteria of each strain. Mice were culled five days post-infection, and the spleens were homogenized in 1 ml of PBS and spread on BCGA. Individual colonies were analysed by PCR with primers specific for each mutation in order to examine the distribution of each strain. Spleens from at least three animals were collected for each pair of strains, and at least 200 colonies were analysed by PCR. The CI was calculated for each strain by dividing the ratio of mutant/wt after infection (determined with PCR) with the ratio of mutant/wt before infection (determined by viable count). Statistical analysis was performed with a GraphPad Prism computer software program using a paired Student's *t*-test (one-tailed) where *P *< 0.05 was regarded as significant.

### Gel electrophoresis and Western blotting

Samples were boiled in the presence of SDS and Β-mercaptoethanol for 5 min and then separated on a 12% acrylamide gel by electrophoresis as described by Laemmli [31]. Proteins were transferred to Immobilon-P Transfer Membranes using a Trans-Blot Semi-Dry transfer cell (BioRad). Membranes were blocked overnight in Tris-buffered saline (TBS) with 5% nonfat dry milk. Membranes were probed with rabbit polyclonal anti-PilA sera [22] and a horseradish peroxidase-conjugated anti rabbit antibody (Amersham Pharmacia Biotech) was used as secondary antibody and the filters were developed by using the ECL Kit (Amersham Pharmacia Biotech) according to the instructions from the manufacturer.

## Authors' contributions

ALF carried out major parts the molecular genetic studies, participated in analysing samples from the animal assay and drafted the manuscript. ENS carried out parts of the molecular genetic studies, participated in analysing samples from the animal assay and drafted the manuscript. IG carried out parts the molecular genetic studies. KK analysed samples from the animal assay and performed the transcriptional analysis. SM carried out parts of the molecular genetics studies. RT was supervising and coordinating parts of the molecular genetics studies. PO supervised and also carried out key parts of the animal work and was involved in supervising the molecular genetics work. LN was involved in analysing bacterial ratios from animal samples and editing of the manuscript. AS supervised the molecular genetics work for parts of the mutagenesis work. ÅF conceived of the study, participated in its design, coordination and helped to draft and edit the manuscript. All authors read and approved the final manuscript.
